# Revegetation re-carbonizes soil: Patterns, mechanisms, and challenges

**DOI:** 10.1016/j.fmre.2024.06.004

**Published:** 2024-06-22

**Authors:** Qingyin Zhang, Yaxian Hu, Mingan Shao, Xiaoxu Jia, Xiaorong Wei

**Affiliations:** a State Key Laboratory of Soil Erosion and Dryland Farming on the Loess Plateau, Northwest A & F University, Yangling 712100, China; b College of Soil and Water Conservation Science and Engineering, Northwest A & F University, Yangling 712100, China; c Key Laboratory of Ecosystem Network Observation and Modeling, Institute of Geographic Sciences and Natural Resources Research, Chinese Academy of Sciences, Beijing 100101, China

**Keywords:** Carbon cycling, Carbon sequestration mechanism, Carbon spatiotemporal variations, Future trends, Revegetation

## Abstract

Revegetation has been widely implemented throughout the world for controlling soil loss, conserving biodiversity, increasing ecosystem productivity, mitigating climate change, and contributing to soil carbon (C) sequestration. However, systematic knowledge is still lacking regarding the variations, mechanisms, and challenges in soil C sequestration after revegetation despite its crucial role in resolving the ongoing C sink/source debate and achieving the C neutrality targets. In this review, we summarize the spatiotemporal patterns and drivers of soil organic carbon (SOC) sequestration in restored ecosystems based on existing studies at multiple spatial and temporal scales and the mechanisms associated with C stabilization in soils after revegetation, and suggest future research into soil C in restored ecosystems. Revegetation, i.e. grassland restoration or afforestation/reforestations, significantly increases the SOC sequestration by 21.4% with rapid SOC accumulation in the initial few years (generally within 30 years), followed by a relatively stable stage. We also clarified the three key mechanisms (including physical protection within soil aggregates, chemical protection by interacting with organo-mineral associations, and inherent biological recalcitrance protection) associated with SOC stabilization in soils after revegetation. Revegetation re-carbonizes soil by increasing new inputs of C and decreasing the ratio of soil C outputs to new inputs of C (i.e., increasing the decomposition of SOC and reducing erosional SOC loss), both of which promote SOC sequestration. Based on the key issues identified in this review, future research should focus on the fate of C sequestrated in soils after revegetation, and feedback to environmental changes and human activities in order to achieve C neutrality by around 2050.

## 1 Introduction

The latest report from the International Panel on Climate Change (IPCC) advocates that global energy systems in both developed and developing countries should strive for carbon (C) neutrality by around 2050 in order to attempt to limit the rise in the global average surface temperature to 1.5 °C [1,2]. Vegetation restoration plays a vital role in achieving C neutrality through fixing atmospheric CO_2_ [3,4]. The annual net C uptake from the atmosphere by terrestrial ecosystems is 2.0 to 3.4 Pg C and it plays critical roles in mitigating climate change [5,6]. Soil is the largest C pool in terrestrial ecosystems and it can have significant impacts on global C cycling and neutrality, especially considering that the amount of soil organic carbon (SOC) (approximately 1,550 Gt in 1-m soil depth) is more than three times the combined amount of C in the atmospheric and biotic pools, and it is also actively exchanged with the other two pools, as well as soil inorganic carbon (SIC) pool [5].

**Fig. 1:**
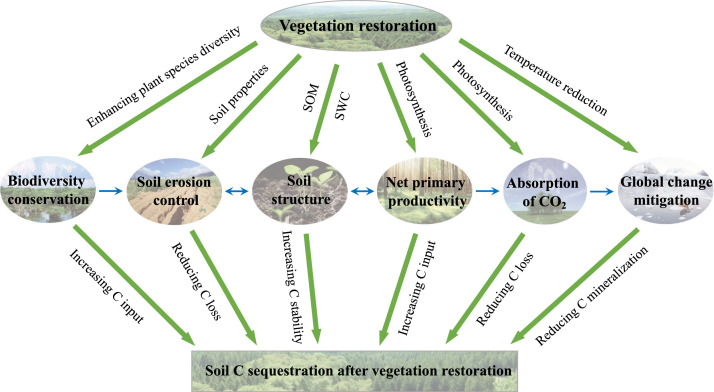
**Benefits of revegetation.** In general, vegetation restoration can enhance biodiversity conservation by increasing the diversity of plant species, improving the soil structure by increasing the soil organic matter inputs and soil water contents, increasing the net primary productivity by enhancing photosynthesis, regulating global climate change by absorbing atmospheric CO_2_ and reducing temperatures, and alleviating soil erosion by improving the soil physicochemical properties. All of these processes can promote the sequestration of soil C by various pathways, such as increasing soil C inputs and reducing soil C losses. SOM: soil organic matter; SWC: soil water content.

Revegetation or vegetation restoration, i.e., establishing long-term plant communities through natural succession, grass seeding (grassland), or tree planting (afforestation or reforestation) on cropland, abandoned land, or degraded land after deforestation, grazing, and other agricultural practices [7,8], is one of the most effective and widely applied approaches for remediating degraded ecosystems [[9], [10], [11]]. This approach evidently increases biodiversity and ecosystem net primary productivity, reduces soil erosion, sequesters C in ecosystems, and mitigates climate change, and these effects usually interact with each other (Fig. 1) [3,12]. All of these effects result in the accumulation of C in soils (usually in organic form, i.e., SOC) by increasing C inputs and/or reducing C losses [3,13]. Therefore, revegetation has great potential for capturing the atmospheric C and mitigating climate change [11,[14], [15], [16]]. Revegetation induces the re-carbonization of soils to provide an important pathway for achieving C neutrality by 2050. However, there is no comprehensive review of this newly sequestrated C in soils after revegetation, e.g., how revegetation re-carbonizes soils and how this newly sequestered C stabilizes in the soil, which leads to uncertainty regarding our understanding of its responses and feedback to environmental changes and human activities [14,17,18].

In this review, we aim to address this issue by summarizing recent advances in our understanding of soil C sequestration after revegetation. First, we describe the current status and general widespread benefits of revegetation for balancing ecological restoration and conservation, land management, and climate change mitigation. We then consider recent research into soil C sequestration after revegetation, including how the effects of revegetation on soil C vary in space and time, the key mechanisms associated with C stabilization in soils after revegetation, and the effects of revegetation on soil C cycling. Finally, we identify some open questions that need to be resolved and suggest challenges that require further investigation in the future.

## 2 General patterns and benefits of revegetation

### 2.1 Current status of revegetation with forest or grassland

Revegetation with trees, i.e., afforestation and/or reforestation, is a major and important vegetation restoration strategy for remediating forests and increasing the global forest area, with significant benefits for ecosystem functionality and soil C sequestration [3,19,20]. To promote forest restoration, the United Nations General Assembly declared 2021–2030 as the United Nations Decade for Ecosystem Restoration, with the global aim of restoring 350 million ha of forests by 2030 [21]. In fact, according to the Global Forest Resources Assessment 2020, the area of planted forests had increased by 123 million ha since 1990, thereby accounting for 3% of the global forest and 45% of the total planted forests. However, the application of afforestation differs markedly among regions and countries. In particular, the forest cover continues to increase in some countries throughout Europe, North America, and China, but it is decreasing in other developing and underdeveloped countries [[22], [23], [24]]. For example, recent satellite data (2000–2017) indicated a striking greening pattern where China alone accounted for 25% of the global net increase in the forest area but with only 6.6% of the global vegetated area [23]. By contrast, the forest area in developing countries decreased from 21.8 to 21.3 million km^2^ from 1992 to 2015, and the rate of decline slowed after 2004 [24].

Revegetation with grassland, i.e., grassland restoration, mainly refers to converting cropland or degraded land into grassland, and both contribute to expanding the global grassland area [25]. It has been reported that global grassland areas decreased between 1990 and 2004 but then increased by 2015, with an average increase rate of 2,900 million ha year^−1^ [26]. Grassland restoration in the USA was accelerated by the passage of the 1985 Farm Bill, which authorized establishment of the Conservation Reserve Program, thereby resulting in the restoration of over 13 million ha of grassland by 2009 [27]. Since 2000, the Chinese Government has launched many major grassland restoration projects, including the Beijing–Tianjin Sand Source Control Project, Returning Grazing Land to Grassland Project, Natural Grassland Protection, Desertification Control, and Grassland Monitoring and Warning, which together have contributed to increasing the area of grassland in China by 4.7% [20].

### 2.2 Benefits of revegetation

The benefits of revegetation projects are not limited to the self-ecosystem. They can also exert positive effects on mitigating climate change, especially in terms of soil carbon sequestration (Fig. 1). Firstly, estimates of the effects of global land-use changes on soil erosion between 2001 and 2012 indicate that revegetation on cropland decreased soil erosion by 12.5 Mg ha^–1^ year^−1^ [28]. The positive effect of revegetation on controlling soil erosion can be ascribed to the increased plant canopy and litter inputs after restoration, which significantly dissipate the energy of rainfall and protect the soil surface from the direct impact of raindrops, thereby preventing the generation of runoff and soil losses [29]. Secondly, revegetation can effectively recover plant, animal, and soil biodiversity (referring to the richness of biological species in the soil, including bacteria, fungi, protozoa, nematodes, insects and other organisms) in degraded ecosystems by creating new habitats [30]. A meta-analysis based on 89 restoration assessments in a wide range of ecosystem types across the globe indicated that ecological restoration increased the provision of biodiversity by 44% [30]. Thirdly, revegetation could also increase ecosystem productivity due to the increases in biodiversity and the abundance of vegetation [31,32]. Globally, the terrestrial ecosystem net primary productivity tended to increase at a rate of 0.288 g C m^–2^ year^–1^ from 2000 to 2015, mainly due to revegetation [32]. Fourthly, revegetation can effectively mitigate global warming by reducing atmospheric CO_2_ concentrations through photosynthesis [3] and cooling the temperature of the earth's surface [33]. In fact, surface cooling by revegetation can be much more effective at mitigating global warming than CO_2_ uptake because revegetation usually promotes evapotranspiration to provide an abundant water vapor supply for the formation of precipitation [34]. Lastly, revegetation significantly increases the amount of C stored in biomass and it eventually leads to an increase in the terrestrial C sink. Global bookkeeping estimates suggest that forest revegetation took up atmospheric C at a rate of 2.5 ± 0.4 Pg C year^–1^ between 1990 and 1999, and then at a rate of 2.3 ± 0.5 Pg C year^–1^ between 2000 and 2007 [6]. In particular, a study showed that forests planted in China accounted for about 80% of the forest biomass C sink since the 1980s [16]. In Central America, revegetation induced the accumulation of SOC at a rate of 13–21 Tg year^−1^, with the potential to accumulate hundreds more Tg of C within a century [11]. There is a general consensus that revegetation re-carbonizes soils but the global pattern of this effect remains unclear. Thus, it is necessary to systematically identify the dynamic variations in climate, biomes, and soils during/after revegetation to facilitate the effective decarbonization of the atmosphere and eventually achieve C neutrality and the Sustainable Development Goals of UN Agenda 2030.

## 3 Spatial and temporal dependence of responses of soil organic carbon to revegetation

### 3.1 Spatial variations in soil organic carbon changes after revegetation: from plot to global scales

**Fig. 2:**
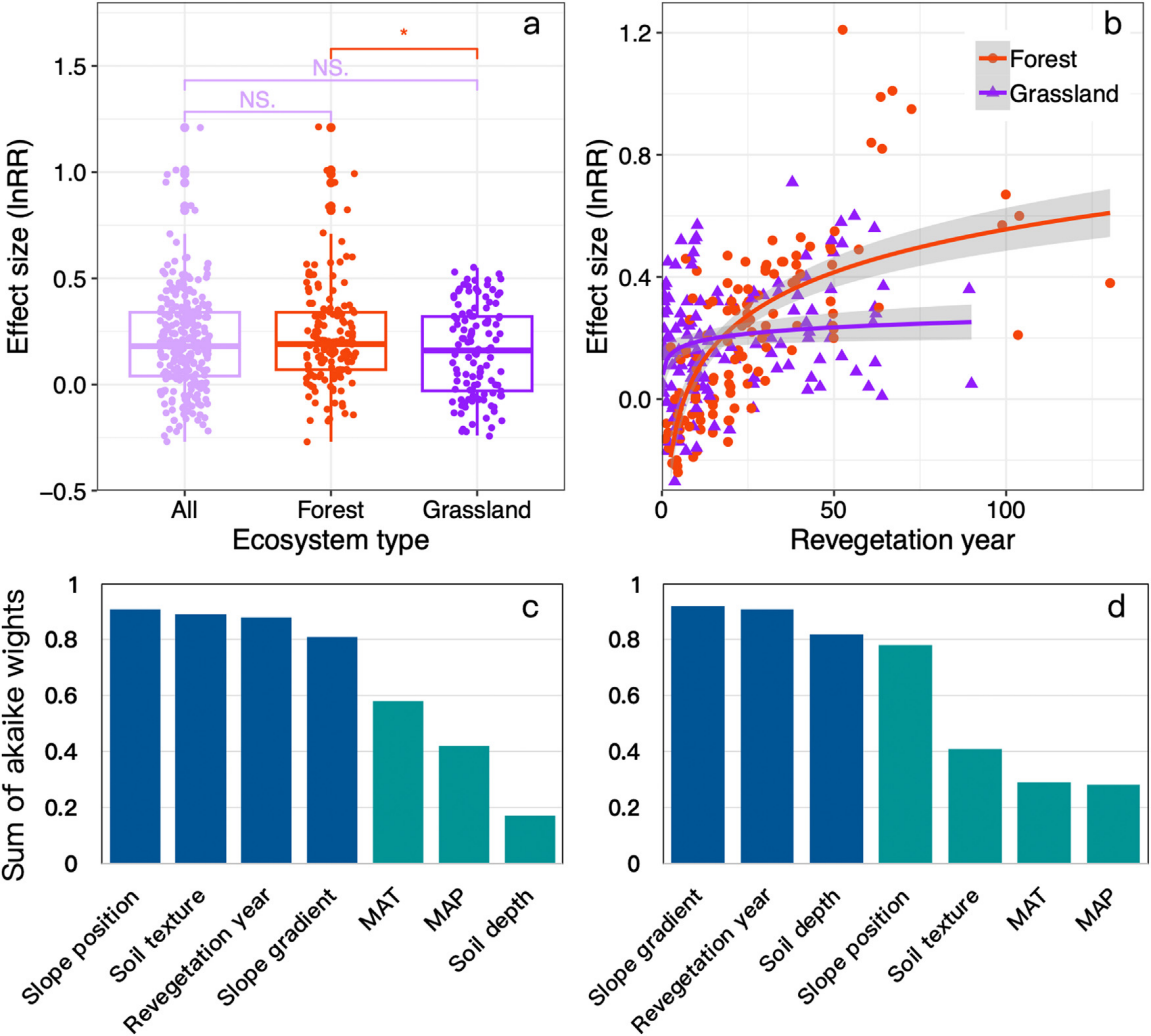
**Spatiotemporal patterns of soil C after revegetation (a, b).** (a) Influence of land use on changes in SOC concentration after revegetation with forest and grassland at plot scale. (b) Relationships between effect size (ln (R)) of SOC concentration and revegetation year. Relative importance of the variables for the responses of SOC to revegetation with grassland (c) and forest (d). The variables include spatial parameters (slope gradient and slope position), soil parameters (soil texture and soil depth), climate factors (i.e., MAT and MAP), and temporal factors (revegetation year). Cutoff is set to 0.8 to estimate important and minor variables.

To elucidate the scale dependency of the SOC after revegetation, we synthesized the results obtained in 88 studies to evaluate the effects of revegetation with forest and grassland on the SOC contents (detailed Materials and Methods were included in Supplementary materials). Compared with adjacent control land (e.g., cropland), revegetation generally significantly increased the SOC concentrations throughout the world, where the effects were significantly greater in forest (+23.3%) than grassland (+16.1%) (Fig. 2a) because a higher ratio of the C allocated to plant biomass is belowground than aboveground in forest compared with grassland [25]. The dominant paradigms hold that the increase in soil SOC contents after revegetation can be attributed to the difference in the magnitude of nutrient input and output fluxes between revegetation and cropland or bare land [35]. In general, the annual harvesting of plant biomass reduces the SOC input to the soil, and agricultural practices (e.g., tillage and irrigation) often induce soil SOC mineralization resulting in greater soil SOC losses [15]. In contrast, revegetation can reduce the soil SOC losses caused by human disturbance, and input more litter into the soil [36], thus increasing the SOC contents, which could explain our partial results and be consistent with the SOC changes (forestland vs cropland: 4.45 g/kg; grassland vs cropland: 3.34 g/kg) studied by Yang et al. [37].

In this meta-synthesis, all the 88 studies represents the SOC changes at the plot or slope scales. We found that the environmental factor of SOC in forest and grassland were highly heterogeneous (Fig. 2c, 2d). For revegetation forest, the main influencing factors of SOC were slope gradient, restoration year, soil depth, and slope position (Fig. 2c); and those of grassland were slope position, soil texture, restoration year, and slope gradient (Fig. 2d). Similarly, many previous studies shown that soil depth, soil texture and restoration year were important factors affecting soil C accumulation in vegetation restoration [35,38]. Furthermore, we found that climate factors, such as MAT and MAP, were less important to the soil C accumulation in forest than were other factors, which was consistent with the results of other studies [[37], [38], [39]]. Therefore, spatial factors should be included as a key factor influencing the SOC contents in revegetation in future studies. Specially, slope characteristics, such as slope position and slope gradient, were the key topographic drivers of soil erosion and nutrient loss [40]. Many researchers noted the importance of slope position and gradient on soil nutrients attributed to soil moisture differences at different slope position and gradient, which could influence the SOC infiltration or leaching [40,41]. In addition, our results indicated that soil texture had greater influence on soil C sequestration after revegetation (especially grassland) (Fig. 2). The reason could be as follows: soil texture significantly affects vegetation (especially herbaceous plant) growth and development [42]. In generally, the medium texture soil has good soil porosity and suitable water conditions, which are conducive to the growth and development of vegetation and higher primary productivity in vegetation restoration [43], and thus the soil C accumulation amounts are higher. However, at the large spatial scales (e.g., regional and global scales), the capacity for SOC sequestration after revegetation is most strongly associated with the climatic conditions [44]. Our results confirmed that humid and hot areas were conductive to SOC accumulation. Although soil organic matter decomposition could be accelerated under high precipitation and temperature conditions, high temperature and precipitation contribute to more litter from plants [45] and increase C inputs to soil, and the dense vegetation coverage reduces the mineralization of SOC by heat and precipitation [35].

### 3.2 Temporal changes in soil organic carbon after revegetation

Determining the temporal changes in SOC after revegetation is essential for evaluating the mechanisms and potential for SOC sequestration in restored ecosystems by providing critical parameters for incorporating SOC cycling into Earth system models. After pooling all of the revegetation types together, SOC increased with the revegetation age in a logarithmic manner, with rapid SOC accumulation in the initial few years (generally within 30 years), followed by a relatively stable stage (Fig. 2b). This nonlinear sequestration of SOC at a global scale is consistent with other studies [11,46,47]. However, grassland and forest showed different increasing trends with revegetation year (Fig. 2b). Specially, the SOC accumulation in grassland significantly increase during the early stage of revegetation, whereas that in forest increased slowly (Fig. 2b). With the increase in revegetation years, forest showed the significantly mounting trend due to the decreasing in human activities [39] and increasing vegetation litter increases and soil macroaggregates increase [48], which were conducive to better accumulation of SOC accumulation. Furthermore, forests have little impact on soil, and soil microbial types and activities and soil physical and chemical properties are mainly affected by the legacy of previous use [49], resulting in the soil C input from forestland being labile. As forest revegetation develops, the input of plant litter increases, the soil environment is gradually stabilized, and the content of SOC begins to increase significantly [50]. Unlike forest, most of the biomass in grassland is concentrated in the underground biomass, of which turn rate is much faster than that in the forest soil environment, thus increasing the vegetation residue and microbial necromass input fluxes in the early stage of revegetation [51]. In addition, the temporal changes in SOC accumulation induced by revegetation are influenced by the ratio of particulate soil organic matter (SOM) relative to mineral-associated SOM fractions [50]. Mineral-associated SOM has a lower average C:N ratio because of its relatively greater microbial origin, longer mean residence time in soils (from decades to centuries) compared with particulate SOM (< 10 years to decades), and stronger chemical bonding with soil minerals and physical protection by fine aggregates [52]. Therefore, mineral-associated SOM contributes to longer-term organic carbon sequestration in soils, thereby leading to relatively stable organic carbon sequestration during the later stage of revegetation.

## 4 Mechanism of carbon stabilization in soils

**Fig. 3:**
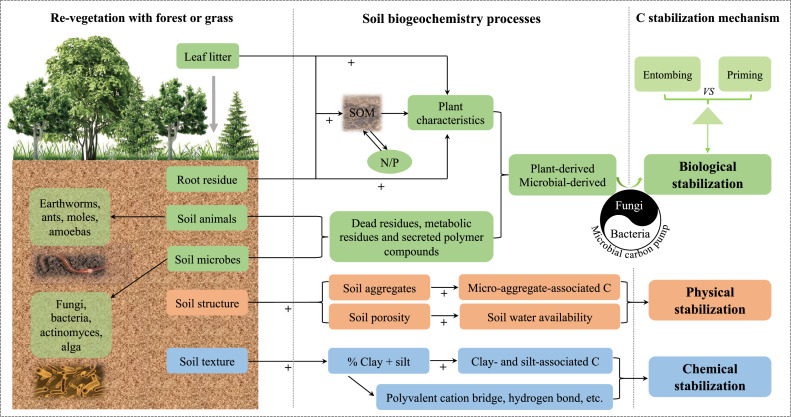
**Conceptual framework showing the distinct mechanisms that allow revegetation to affect soil C sequestration, including the relationships between the benefits (plant litter, soil animals, soil microbes, and soil properties) of revegetation and soil C stabilization mechanism.** (1) In the physical mechanism, revegetation usually improves the soil structure, such as increasing the fractions of macro- and micro-aggregates in soils to enhance the physical protection of new C inputs and the original C. (2) In the chemical mechanism, revegetation usually increases the soil C inputs via the association of C with soil minerals (silt and clay particles), such as through ligand exchange, polyvalent cation bridges, and hydrogen bonding. (3) In the biological mechanism, revegetation promotes the growth of soil animals and microorganisms, which also play active roles in the decomposition of plant residues. Plant-derived SOM can fuel the microbial carbon pump (Liang et al. [82]) to drive the accumulation of microbial necromass, which is preferentially preserved in the mineral-associated SOM and contributes to the stabilization of C in soils. The *yin-yang* symbol in the microbial carbon pump image is used to illustrate the conversion of C to microbial necromass, which is driven in different ways by both bacteria and fungi with different trophic strategies. SOM: soil organic matter; N: soil total nitrogen content; P: soil total phosphorus content.

Soil C stabilization, i.e., the ability of soil C to resist loss, is mainly controlled by three key mechanisms: physical protection within soil aggregates, chemical protection by interacting with organo-mineral associations, and inherent biological recalcitrance protection [53] (Fig. 3). These mechanisms can interact with each other (e.g., aggregations of organo-mineral associations can already include biochemically recalcitrant SOC) and their respective contributions to SOC stabilization are strongly influenced by the inherent soil properties and external environment [54,55].

### 4.1 Physical protection

The primary mechanism responsible for the physical protection of new soil C inputs involves incorporating SOC within aggregates [53,56] and protecting SOM by forming physical barriers between microbes, enzymes, and their substrates. The compartmentalization of SOC and microbes can also reduce oxygen diffusion to inhibit the microbial activity within aggregates [57]. These effects tend to be more pronounced for micro-aggregates (usually 0.053–0.25 mm) than macro-aggregates (> 0.25 mm) due to the greater density and lower porosity of the former [58]. A study using the ^13^C tracing technique showed that the average turnover time for C was 412 years in free micro-aggregates but only 140 years in macro-aggregates in surface layer soils [56]. Macro-aggregates (> 0.25 mm) cannot directly provide long-term protection for C but they often contain more C than micro-aggregates, and thus they can potentially play a vital role in the C sequestration process [53].

### 4.2 Chemical stabilization

The chemical stabilization of new C inputs in soils is dominated by the association of C with soil minerals (silt and clay particles), including via ligand exchange, polyvalent cation bridging, hydrogen bonding, chelation, and hydrophobic interactions [59]. SOC was found to have significant positive correlations with the clay contents in various ecosystems [55], mostly because silt- and clay-associated C has a longer turnover time than C in other larger soil particles (e.g., the sand fraction) [60]. The SOM retained in fine soil fractions is highly decomposed and strongly associated with clay minerals and pedogenic oxides, whereas the particulate SOM in coarse fractions mainly comprises plant residues, roots, and other plant debris at various stages of decomposition [61,62]. Polyvalent cations such as Ca^2+^, Fe^3+^, and Al^3+^ bridge SOM molecules with clay particles and contribute to organo-mineral associations. For example, Ca^2+^ plays an active role in stabilizing SOC by inner- and outer-sphere bridging, as well as stabilizing soil particle-occluded SOC by enhancing the aggregation of soil particles [63]. A significant amount of mineral-associated C is adsorbed or co-precipitated with Fe minerals due to their high surface area (< 5% of the total surface area of soil sediments) and ubiquity in soils [64]. In addition, the association of C with Fe oxides is affected by the soil pH, which regulates the propensity of SOC to bind with Fe oxides [65].

### 4.3 Biological mechanisms

The biochemical stabilization or protection of new soil C inputs is due to the complex chemical composition of organic materials, and this inherent property of SOM renders it more resistant to subsequent decomposition [59]. The formation of biostable C pools occurs via the selective retention of primary and secondary refractory products (macromolecules) derived from plants and soil biota, i.e., primary and secondary anti-decomposition substances. The primary anti-decomposition substances include polymers in plants that are difficult to degrade due to their aromatic ring structures, such as lignin, and macromolecules that contain multi-alkyl compounds, such as lipids, waxes, keratin, and suberin [66]. Many laboratory and field experiments have demonstrated that these plant components are difficult to degrade and utilize, and thus they gradually accumulate in soil to become part of the stable SOC pool [67]. In general, about 41%, 40%, and 20% of the photosynthetically assimilated C enters the roots in forest, agricultural, and grassland ecosystems, respectively [68], and most C is stabilized in soil.

The secondary anti-decomposition substances are refractory metabolites formed by soil animals and microorganisms during the decomposition of plant residues, including dead residues, metabolic residues, and polymer compounds secreted by soil animals and microorganisms [[69], [70], [71]]. Soil animals, such as earthworms, are considered key engineers that may affect decomposition by mixing and simultaneously ingesting fresh organic matter and minerals [70]. Earthworms can enhance the decomposition of C by digesting and transforming materials in their guts, as well as stabilizing SOM through the formation of new aggregates [72]. These new aggregates enhance the physical protection from microbial mineralization for new C inputs induced by revegetation for years to decades [73]. Some studies have shown that earthworms could destroy the mycelium of the fungus and reducing its abundance, resulting in enhancing the transformation of fresh SOM into microbial necromass to increase the stabilization of C [67,74]. In addition to animals, soil microorganisms have critical but contrasting roles in controlling terrestrial C fluxes, where they can promote the release of C into the atmosphere through their catabolic activities, but also increase C inputs by stabilizing C in a form that is not readily decomposed [75].

### 4.4 Carbon stabilization in soils after revegetation

**Fig. 4:**
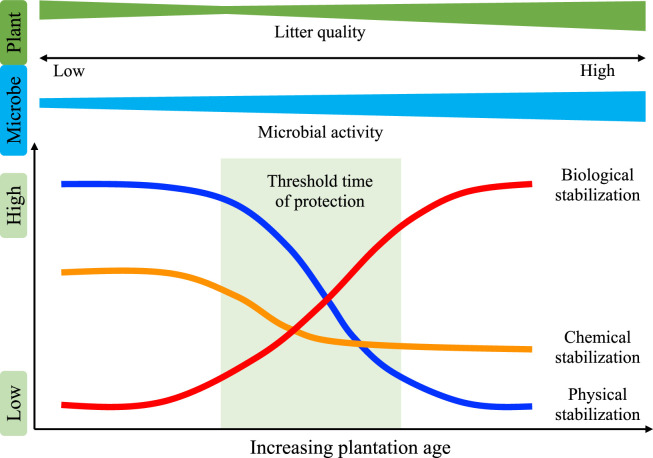
**Conceptual diagram depicting the potential variations in three mechanisms responsible for C stabilization in soils after revegetation.** The physical and chemical stabilization of C is mainly determined by the association with C, but the contributions to C stabilization in soils may decrease during the revegetation process due to the decreased number of available sites for minerals to associate with C, and the aggregation of soil particles may reach equilibrium or saturation. The biological protection of C in soils may increase during the revegetation process due to the increased microbial activity, improved litter quality, and more suitable habitats for soil biota. However, the threshold times when shifts occur should be identified.

After re-carbonization due to revegetation, soil C must be persistently stabilized before it can effectively resist perturbation (resistance) or recover from perturbation (resilience) [76,77]. Revegetation usually increases the fractions of macro- and micro-aggregates in soils to enhance the physical protection of new C inputs and the original C during the first few years [60,78]. For example, in China’s Loess Plateau, the OC associated with macroaggregates accounted for 76.5%–84.0% of the new OC in bulk soils along the 10–35 years revegetation with forest [78]. In addition, the accumulation of divalent cations (exchangeable Ca and Mg) in soils after revegetation promotes the early formation of large macro-aggregates [79]. However, the aggregation of soil particles may reach equilibrium in the later process and the contribution of physical protection may decrease. Therefore, the physical protection of C on soil aggregates might dominate C stabilization during the early stage of revegetation, but then shift during revegetation over time (Fig. 4). The chemical stabilization of C is mainly determined by the association of C with clay minerals but the contribution of this process to C stabilization in soils may decrease over time during revegetation because of the decreased availability of sites for minerals to associate with C, before finally reaching an equilibrium or saturation status (Fig. 4).

Revegetation can also increase the production of root litter and rhizodeposition, such as sloughed root cells, passive exudation, and active secretion [80], thereby leading to the proliferation of soil microbes in the rhizosphere. These processes enhance the accumulation and stabilization of soil C in several ways. In particular, plant root litter inputs containing high amounts of chemically recalcitrant components, such as lignin, may promote the formation of particulate SOM and enhance the short-term stabilization of soil C [81]. Alternatively, the labile substrates from root exudates after revegetation may stimulate microbial growth to fuel the “microbial carbon pump” [82], which could drive the accumulation of microbial necromass and extracellular compounds that are preferentially preserved in the mineral-associated SOM, and contribute to the stabilization of C in soil [82,83]. In addition, associated microbes such as mycorrhizal fungi play vital roles in the aggregation and structural development of soil [80]. For instance, the root-derived extracellular polymeric substances and fungal hyphae associated with roots may enhance soil aggregation. Nevertheless, it is still unclear how arbuscular mycorrhizal fungi and ectomycorrhizal fungi might affect the overall storage and persistence of SOC after revegetation, and thus it is necessary to elucidate the mechanisms responsible for regulating the effects of mycorrhizae on soil C dynamics, and their control after revegetation with grassland and forest. In addition, revegetation usually increases soil C inputs, improves the soil structure, alleviates soil environment stresses, and provides a more suitable habitat for soil biota [69], thereby contributing to the increased biological stabilization of soil C in restored ecosystems. Hence, the biological stabilization of C in soils should increase over time with revegetation and identifying the time when a shift occurs is critical for assessing the role in stabilization (Fig. 4).

## 5 Effects of revegetation on soil carbon cycling pathways

### 5.1 Increases in soil carbon inputs

Revegetation leads to the accumulation of organic C in soil by increasing new C inputs [84,85]. A study based on the ^13^C tracing technique showed that new SOC inputs increased during the early afforestation stages (< 10 years), followed by a relatively stable state [78]. In general, the composition, structure, and diversity of vegetation are relatively simple during the early stage of restoration, with low plant residue and root exudate inputs [86]. As the revegetation process continues over time, plant communities become more diverse and stable, and plant residue inputs increase [86,87]. Furthermore, changes in the soil physicochemical properties after restoration can affect the decomposition and accumulation of SOC [55]. The improved soil structure enhances the association of new C inputs with soil particles and C sequestration in soils. For example, the accumulation of organic C in soil after afforestation mainly occurs in macro-aggregates in various regions [88]. In addition, decreased harvesting or not harvesting biomass after revegetation promotes the accumulation of SOC [84].

**Fig. 5:**
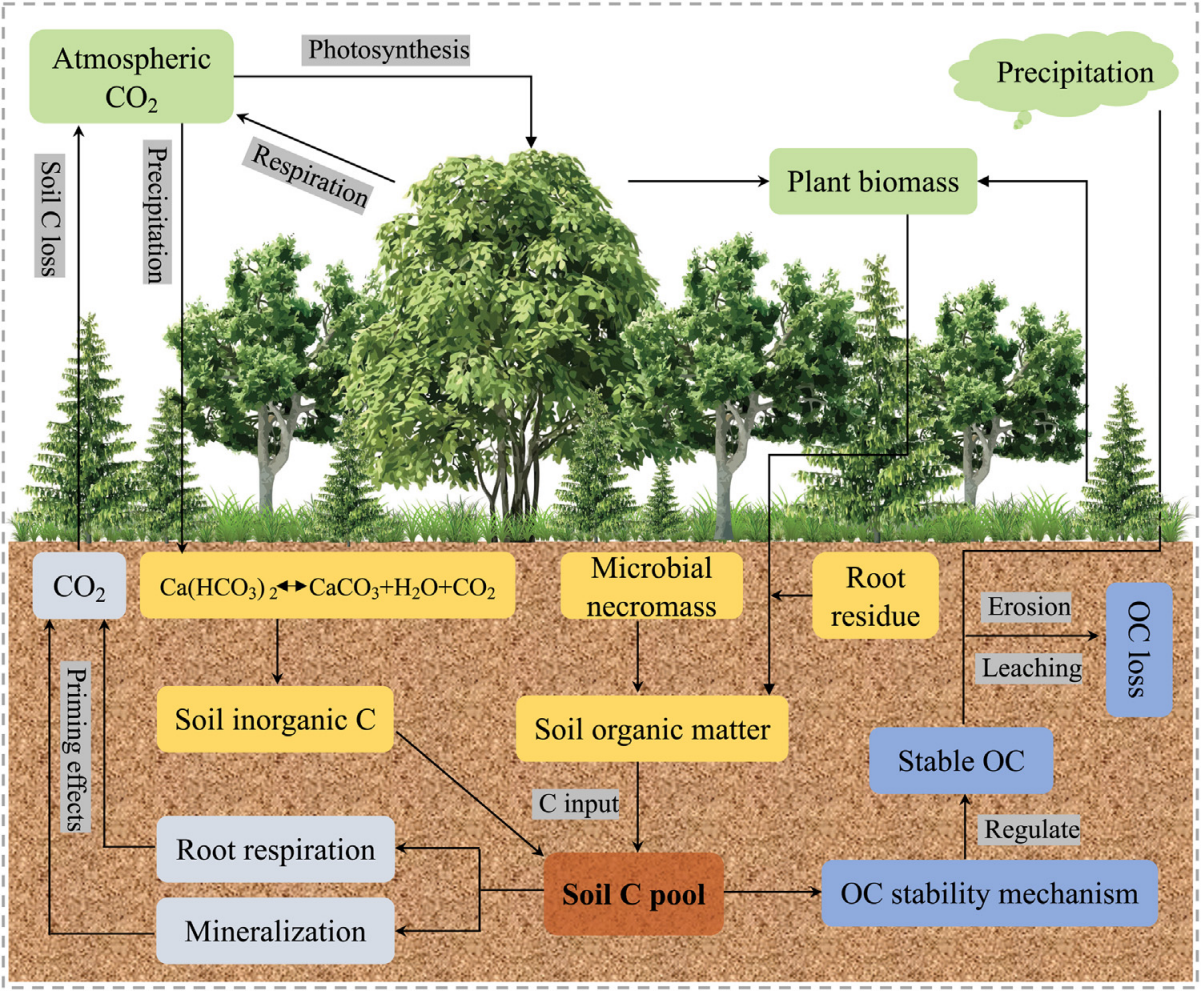
**Conceptual framework showing the effects of revegetation on soil C cycling, including soil C inputs, C outputs, soil inorganic C, and C turnover.** During revegetation, plant communities become more diverse and stable, and plant residue inputs increase. In addition, biotic interactions between soil microorganisms and animals are the primary C-containing components that contribute to the stable SOC pool after revegetation. Revegetation can also decrease soil C outputs through the reduced decomposition of SOC and net effects of soil erosion. However, the effect of revegetation on soil inorganic C is uncertain because of variations in SOC and pedological factors, such as the sand + silt contents and pH. All of these processes can prolong the soil C turnover time and contribute to the sequestration of C in soils.

Biotic interactions between soil microorganisms and animals have essential roles in regulating soil C inputs [89]. Recent studies showed that fungal and bacterial necromass are the primary C-containing constituents that contribute to the stable SOC pool after revegetation [71] (Fig. 5). Thus, the main driver of SOC accumulation under some conditions might not be the decomposition and transformation of litter *per se*, but instead microbial growth could lead to the deposition of microbial-derived C in the soil C reservoir via biomass turnover and the accumulation of necromass after revegetation [90]. Moreover, soil animals (e.g., earthworms) are known to be important for aggregating particles, soil C inputs, and stabilization [91]. A study based on ^13^C-labeling clearly indicated that the direct involvement of earthworms in providing inputs and protecting C in microaggregates within large macroaggregates possibly led to the long-term stabilization of soil C [89].

### 5.2 Decreases in ratio of soil carbon outputs to carbon inputs

The pathways responsible for C loss from soils mainly involve the decomposition of SOC, which may be controlled by two contrasting mechanisms in restored ecosystems. In particular, revegetation usually increases the litter inputs but also the decomposition of organic matter and activities of soil microorganisms, which increase root and microbial respiration [92]. The supply of fresh plant-derived C after revegetation into the soil may accelerate the decomposition of SOC and decrease soil C stocks (known as the “priming effect”), which can increase SOC decomposition by up to 350% compared with soils without new C inputs [93]. However, the proportion of C lost through respiration relative to the total C input usually decreases after revegetation, thereby leading to a net increase in the SOC pool [94]. In addition, the soil temperature is the key factor that influences the decomposition of SOC but it usually decreases after revegetation due to the increased soil water content and litter cover on the land surface, and thus the decomposition of SOC is reduced [95]. It is acknowledged that C decomposition increases with the soil temperature, but revegetation significantly decreases the local air temperature and soil temperature, as well as their seasonal variations [84,96]. These effects could also greatly decrease the response of C decomposition to temperature change and reduce soil C losses from restored ecosystems more than expected in a warming world. Moreover, the decomposition of litter can contribute organic matter to soils, improve the soil structure and permeability, and reduce the horizontal surface runoff velocity to decreases SOC losses [97].

The net effects of erosion on soil C cycling remain among the most widely debated uncertainties when attempting to quantify the potential of soils for mediating climate change [98], but the contribution of revegetation to reducing erosional soil C losses has been clearly demonstrated in both experimental and modeling studies [29]. For instance, Shi et al. [99] reported that revegetation significantly inhibited soil erosion and subsequent C losses by reducing sediment production and runoff in a field study. By using the Revised Universal Soil Loss Equation (RUSLE), Gou et al. [100] found that the amount of SOC that underwent substantial lateral redistribution due to soil erosion was 10.46 Tg C yr^−1^ during the period of 2000–2017, which was a decrease of 21% after vegetation restoration. In summary, considering the slight increases in the decomposition of SOC and reduced erosional soil C losses, the ratio of soil carbon outputs to carbon inputs ultimately decreases after revegetation (Fig. 5).

### 5.3 Uncertainties regarding the changes in soil inorganic carbon

Inorganic C, including lithogenic inorganic C and pedogenic inorganic C, accounts for a large fraction of C in soils, particularly in arid and semiarid regions, and it comprises approximately one-third of the global soil C pool [101]. Soil inorganic C (SIC) plays an important role in C sequestration and climate mitigation through the formation of pedogenic SIC or carbonate dissolution, and the changes in SIC usually interact with those in SOC [102]. Hence, determining the changes in SIC after revegetation is essential for understanding soil C cycling.

At present, the effects of revegetation on SIC remain uncertain, where decreases [103], increases [104], or no changes in SIC have been reported after revegetation [105]. In particular, the accumulation of SOC and root exudates after revegetation can stimulate soil respiration and the accumulation of CO_2_ in soil air spaces to promote carbonate dissolution and HCO_3_^−^ production [102]. This process further promotes carbonate precipitation [106] to increase SIC in restored ecosystems and enhance the sequestration of C in soil. However, SIC was reported to decrease significantly in the 0–80 cm soil depth after grassland restoration (i.e., by excluding grazers) in a semiarid region of northwest China [103]. Hussain et al. [105] also found that SIC was higher in degraded land than restored forest in Northern India. The decreases in SIC in restored ecosystems might be explained by the accumulation of SOM generally decreasing the soil pH, thereby resulting in the loss of SIC [107]. In addition, the enhanced plant uptake of Ca^2+^ and Mg^2+^ after revegetation could inhibit carbonate precipitation to reduce SIC [103]. The variations in SOC mainly influence the response of SIC to revegetation in the topsoil [105], whereas pedological factors (e.g., sand + silt contents and pH) are most important in the subsoil [106]. Moreover, changes in the soil moisture, fine root biomass, and SOM in restored ecosystems can further drive the translocation of carbonate within the soil profile [108]. Therefore, the effects of revegetation on SIC should be explicitly quantified and incorporated into C cycling models.

### 5.4 Soil carbon turnover

Soil C turnover is often quantified as the mean residence time, which is defined as the average time that C resides in a pool at steady state or the average time required to completely renew the pool at steady state [53]. A longer turnover time indicates that C is stable in soil. Globally, the average turnover time for SOC in the top 1 m soil depth was estimated as 10.8–39.3 years [109]. Revegetation can prolong this turnover time in the following ways: (1) by increasing the total C input and reducing C losses [95]; (2) by promoting soil aggregation, which facilitates the preservation and stabilization of SOC [110]; and (3) the decrease in the bulk density and increase in soil moisture after revegetation can also inhibit the decomposition of soil C [111]. It should be noted that the climate, vegetation type, and pedological factors have significant effects on C turnover after revegetation [112,113]. However, several knowledge gaps must be addressed in order to obtain reliable evaluations of the soil C dynamics and C cycling–climate feedback.

## 6 Key issues regarding future research in restored ecosystems

According to previous studies, soil C cycling comprising C inputs, outputs, and the turnover of C in soils is evidently influenced by land-use change after revegetation. Thus, future research should focus on the fate of C sequestrated in soils after revegetation, and feedback to environmental changes and human activities in order to achieve C neutrality by around 2050. In the following, we suggest several key issues that should be prioritized for future research to improve our understanding of the effects of revegetation on soil C storage.

### 6.1 Strengthening the research of inorganic carbon in restored ecosystems

Most previous studies have focused on SOC, since it can quickly respond to the climate or revegetation [78,114]. However, few studies focused on SIC, which dominate soil C in such arid and semi-arid regions accounting for 2/5 of the global land area. The SIC pool within 1 meter of the soil profile ranges from 695 Pg to 1,738 Pg, which is about 1/3 of the terrestrial soil C pool [5]. Therefore, SIC pool has a significant impact on the global C cycling. In the past, it was thought that SIC was inactive and the cycling period can be a thousand years or more [115]. However, recent studies have found that higher soil CO_2_ concentrations can accelerate the rate of SIC cycling [116]. Moreover, SIC content and profile distribution were also significantly affected in the short term (at a time scale of several decades) [105], indicating that SIC could rapidly respond to land use changes such as revegetation. Therefore, the effects of revegetation on SIC should be explicitly quantified and incorporated into C cycling models in the future.

### 6.2 Interactions between soil carbon in restored ecosystems with management and environmental changes

**Fig. 6:**
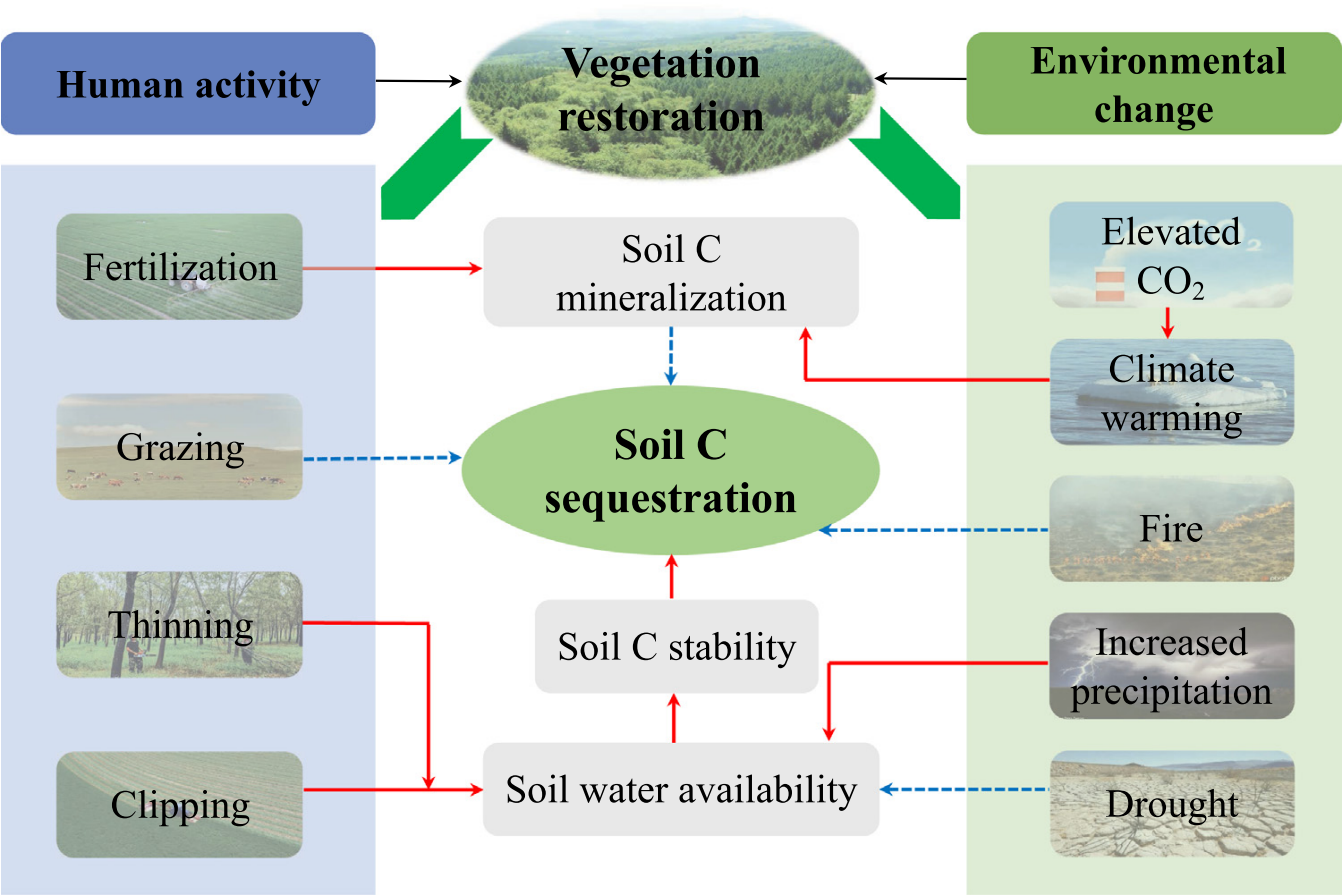
**Conceptual framework showing the feedback between soil C in restored ecosystems with human activities and environmental changes.** The red solid lines indicate increases and the blue dotted lines indicate decreases relative to the next process. In general, human activities such as fertilization, thinning, grazer exclusion, and clipping can increase the C pools by improving the soil structure and soil water availability. Elevated atmospheric CO_2_ and climate warming can promote soil C mineralization, which is detrimental to soil C sequestration. In addition, fires and droughts have profound and disadvantageous impacts on soil C in restored ecosystems. However, it is important to determine how restored C interacts with environmental changes and human activities at different spatiotemporal scales.

Revegetation significantly increases C in soils but the new C inputs are sensitive to ecosystem management and environmental changes. In particular, ecosystem management methods, such as fertilization, thinning, grazer exclusion or enclosing, and clipping, are often used to improve the sustainability and functionality of restored ecosystems [117]. Moreover, terrestrial ecosystems have been affected by remarkable environmental changes (e.g., elevated atmospheric CO_2_, climate warming, drought, extreme precipitation regimes, and fires) over the past few decades [1,3,118], which have profoundly impacted the soil C in restored ecosystems, and thus the feedback between the newly sequestered C and climate change (Fig. 6). However, the changes in soil C are not always synchronized with plant C in response to environmental changes, thereby making it difficult to predict and manage C in restored ecosystems. Understanding how restored C interacts with environmental changes and management methods at different spatiotemporal scales will help to disentangle the underlying connections.

### 6.3 Coupling relationships among carbon, nutrients, and water cycling

The soil C, nutrient, and water cycles are strongly coupled in ecosystems. In particular, the ecosystems in arid and semiarid climates are limited by water, and revegetation depletes the soil water and increases water limitation [119], thereby altering the terrestrial water cycle and the availability of soil moisture required for the uptake of CO_2_ by plants [120]. These changes in water availability could significantly affect the growth of vegetation and enhance or decouple the C–water relationship, with profound effects on predictions of C and water cycles. Furthermore, most terrestrial ecosystems are limited by nutrients [121,122] and revegetation affects their availability to alter their limitation in restored ecosystems [123]. In general, the accumulation of C in soils leads to an increase in N [124,125], which might alleviate the N limitation in soils, and thus the coupling of C–N might be tighter in restored ecosystems than non-restored ecosystems. However, revegetation, particularly with legume plants, may decrease the soil P and enhance the P limitation in restored ecosystems [122]. Therefore, attempts to enhance soil C sequestration via revegetation must consider and assess the water and nutrient demands due to plant growth to minimize the potential microbial priming effect associated with soil C turnover, particularly for ecosystems in arid and semiarid regions limited by water availability.

### 6.4 Modeling the fate of soil carbon in restored ecosystems

Experimental observations provide important direct evidence and a mechanistic understanding of the changes in soil C after revegetation at relatively smaller spatial and temporal scales. However, the integration of this knowledge into terrestrial C models may lead to different predictions and the observations can be poorly fitted [126]. For instance, the 5th Climate Model Intercomparison Project assessed 11 Earth system models and found that none could accurately predict soil C patterns across the global land surface compared with the Harmonized World Soil Database [127], mainly because experimental observations cannot be linearly extrapolated to quantify the global-scale sensitivity of terrestrial ecosystems to climate change over various time spans ranging from decades to centuries. Given the profound impacts of revegetation on soil C cycling and the vast revegetated area throughout the world, integrating some key processes that control soil C cycling (e.g., microbial processes) and the corresponding related factors into traditional Earth system models could be an option for improving the accuracy of C cycling prediction in both soils [126] and terrestrial ecosystems [99].

### 6.5 Innovative soil carbon accounting methods and indicators for restored ecosystems

Conducting soil C sink accounting in a “measurable, reportable, and verifiable” manner is an important scientific basis for policy making guidance by the IPCC for reducing emissions and sink enhancement. The methods used for estimating the C budget in regional restored ecosystems include the inventory method, eddy covariance method, ecosystem process modeling method, and atmospheric inversion method, which have different strengths, weaknesses, and sources of uncertainty [128]. Therefore, uncertainty assessment is a grand challenge at present due to the multiple scales and sources of uncertainties involved. Given that the advantages and disadvantages of these methods are complementary, we suggest that it is necessary to establish a C accounting system based on “field investigations, multiple data types, multiple processes, and model simulations.” In addition, more attention should be paid to accurately understanding the responses of ecosystem C sinks to extreme climate events [129], especially in fragile ecosystems and hotspots (such as the Qinghai–Tibet Plateau, Loess Plateau, and Southwest Karst Region in China) [130], including in the shallow and deep soil layers. Solving these problems can directly facilitate achieving C neutrality and the Sustainable Development Goals of UN Agenda 2030.

### 6.6 Managing re-carbonized soils in restored ecosystems

Manipulating revegetation for soil C sequestration is an appealing strategy. However, at the global scale, restored ecosystems lack high and stable SOC storage levels due to the elevated ecosystem C turnover rate [131]. Thus, the soil C in restored ecosystems should be carefully managed because the fragile restored ecosystems in arid/semiarid or deserted regions are particularly sensitive to climate changes and human activities, and many restored ecosystems are planned for re-use and they may be subject to further disturbance. Therefore, integrated techniques should be developed, assessed, and adopted to increase the C sequestration potential, but also to ensure the stabilization of the newly sequestered soil C in restored ecosystems in order to protect re-carbonized soils. These aims are particularly important when attempting to strengthen the resilience of soil C to human disturbances and environmental changes.

## Declaration of competing interest

The authors declare that they have no conflicts of interest in this work.

## Appendix Supplementary materials

Image, application 1
