# Supplementary material for: Revegetation re-carbonizes soil: Patterns, mechanisms, and challenges
Source: Fundam Res. 2024 Jun 22;6(1):290–300. doi: 10.1016/j.fmre.2024.06.004 (PMC12869759; doi:10.1016/j.fmre.2024.06.004)
Supplement: Supplementary file 1 [file mmc1.docx]

**Supplementary materials**

**Materials and methods**

**Data collection**

To understand which factors drive soil organic carbon (SOC) sequestration dynamics following revegetation across different spatial scales and its temporal pattern, we searched all available peer-reviewed papers through the Web of Science (WOS), Chinese National Knowledge Infrastructure (CNKI), and Google Scholar search engines. The keywords of the collected papers were as follows: “revegetation”, “vegetation restoration”, “land use”, “land use change”, “soil organic carbon”, and “soil physical and chemical properties”. To eliminate unqualified literature, avoid publication deviation and ensure the completeness and scientific quality of each study, we collected papers related to the research topic as much as possible but also examined the references in the papers and other related papers from the same author/research team.

To minimize publication bias, we used the following criteria to select studies: (1) the SOC content should be examined; (2) the paper must have a control group (cropland or bare land) and at least one experimental group (vegetation), and the vegetation types must be clear, including grassland and forestland; (3) the control and experimental group were established with the same initial environmental and climate conditions, soil parameters, and species composition; (4) the means, standard errors (Se) or standard deviations (S), and number of replicates (n) of selected variables in the control and experimental groups could be extracted directly from tables, digitized graphs, or contexts.

In total, 88 peer-reviewed papers were selected, including 690 paired datasets of SOC content. The following indicators were included in this paper: (1) research site (longitude, altitude); (2) climate (MAP, MAT); (3) land-use type (cropland, bare land, grassland, forest) and vegetation plantation time; (4) soil texture, slope gradient, slope position, and soil depth; and (5) runoff, soil loss, SOC content, and soil total nitrogen content.

**Data analysis**

Regional or global studies have used approximate soil bulk density values to estimate SOC stocks, and there are differences in the estimation methods, which overestimate or underestimate soil SOC storage (Barcena et al., 2014; Liu et al., 2014). Therefore, our research used the SOC content data. If neither the standard deviation (S) nor the standard error (Se) of the research report was provided, 1/10 of the mean was substituted (Gattinger et al., 2012; Xu et al., 2019). If the provided data included the Se, S, and n, the was calculated using the following equation:

$S=\sqrt{n}\times S_{e}$ (1)

The natural log-transformed response ratio (lnRR) was used to reflect the effects of different revegetation types on the SOC contents, and it was calculated by the following equation (Gurevitch et al., 1999):

$lnRR=ln(\frac{\bar{X}_{t}}{\bar{X}_{c}})$ (2)

where $\bar{X}_{t}$ and $\bar{X}_{c}$ are the means of the experimental and control pairs, respectively.

The mean, S or Se, and sample size *n* were extracted for each group to calculate the variance (*v*) of the logarithmic effect size as follows.

$v=\frac{S_{t}^{2}}{n_{t}\bar{X}_{t}^{2}}+\frac{S_{c}^{2}}{\bar{X}_{c}^{2}}$ (3)

The weighting factor ($w=1/v$) for each observation was calculated as the weight of the variance. Some studies presented more than two types of observations, so we adjusted the weights by the total number of observations per study. The weighted RR (RR_++_) was calculated using the total weighting factor ($w^{'}=w/n$) according to the following equation:

$lnRR^{'}=w^{'}lnRR$ (4)

${RR}_{++}=\frac{\sum_{i} \ln{RR'}_{i}}{\sum_{i} {w'}_{i}}$ (5)

where $ln{RR'}_{i}$ is the weighted effect size of *i* and ${w'}_{i}$ is the total number of observations of *i* per study.

To determine whether revegetation with grassland or forest had significant effects on the SOC content, the 95% confidence intervals (CIs) were calculated for all analyses. The effect of a global change driver on a variable was significant if the 95% CI value of *RR_++_* did not overlap with zero; otherwise, the global change driver had no significant impact on the variable. Finally, the effect size was converted into the percentage change of a variable (Z) as follows:

$Z=\left[ \exp\left( {RR}_{++} \right)-1 \right]\times100\%$ (6)

In addition, we analyzed the relative effects of multiple factors (i.e., MAP, MAT, slope position, slope gradient, soil depth, soil texture and restoration age) on SOC sequestration in forest and grassland restoration to determine the relative importance of the factors. The relative importance was calculated as the sum of the Akaike weights for all the models in which the factors were included. All variables were tested for normality before statistical analysis. R software was used for the meta-analysis.

References:

1. Barcena, T., Kiaer, L.P., Vesterdal, L., Stefansdottir, H., Gundersen, P. and Sigurdsson, B.D., 2014. Soil carbon stock change following afforestation in Northern Europe: a meta-analysis. Global Change Biology, 20, 2393–2405.
2. Gattinger, A., Muller, A., Haeni, M., Skinner, C., Fliessbach, A., Buchmann, N., Maeder, P., Stolze, M., Smith, P., Scialabba, E.H., 2012. Enhanced top soil carbon stocks under organic farming. Proceedings of the National Academy of Sciences 109, 18226–18231.
3. Gurevitch, Jessica, Hedges, V., L, 1999. Statistical issues in ecological meta-analyses. Ecology 80, 1142–1149.
4. Liu, C., Lu, M., Cui, J., Li, B., Fang, C., 2014. Effects of straw carbon input on carbon dynamics in agricultural soils: a meta-analysis. Global Change Biology, 20, 1366–1381.
5. Xu, H., Sieverding, H., Kwon, H., Clay, D., Stewart, C., Johnson, J.M.F., Qin, Z., Karlen, D.L., Wang, M., 2019. A global meta-analysis of soil organic carbon response to corn stover removal. GCB Bioenergy 11, 1215–1233.
